# Outcome of primary resurfacing hip replacement: evaluation of risk factors for early revision: 12,093 replacements from the Australian Joint Registry

**DOI:** 10.3109/17453671003685434

**Published:** 2010-03-31

**Authors:** Gareth H Prosser, Piers J Yates, David J Wood, Stephen E Graves, Richard N de Steiger, Lisa N Miller

**Affiliations:** ^1^Perth Orthopaedic Institute, Fremantle Hospital and University of Western Australia; ^2^Australian Orthopaedic Association National Joint Replacement Registry; ^3^Data Management and Analysis Centre, University of AdelaideAustralia

## Abstract

**Background and purpose:**

The outcome of modern resurfacing remains to be determined. The Australian Orthopaedic Association National Joint Replacement Registry (AOANJRR) started collection of data on hip resurfacing at a time when modern resurfacing was started in Australia. The rate of resurfacing has been higher in Australia than in many other countries. As a result, the AOANJRR has one of the largest series of resurfacing procedures. This study was undertaken to determine the results of this series and the risk factors associated with revision.

**Patients and methods:**

Data from the AOANJRR were used to analyze the survivorship of 12,093 primary resurfacing hip replacements reported to the Joint Replacement Registry between September 1999 and December 2008. This was compared to the results of primary conventional total hip replacement reported during the same period. The Kaplan-Meier method and proportional hazards models were used to determine risk factors such as age, sex, femoral component size, primary diagnosis, and implant design.

**Results:**

Female patients had a higher revision rate than males; however, after adjusting for head size, the revision rates were similar. Prostheses with head sizes of less than 50 mm had a higher revision rate than those with head sizes of 50 mm or more. At 8 years, the cumulative per cent revision of hip resurfacing was 5.3 (4.6–6.2), as compared to 4.0 (3.8–4.2) for total hip replacement. However, in osteoarthritis patients aged less than 55 years with head sizes of 50 mm or more, the 7-year cumulative per cent revision for hip resurfacing was 3.0 (2.2–4.2). Also, hips with dysplasia and some implant designs had an increased risk of revision.

**Interpretation:**

Risk factors for revision of resurfacing were older patients, smaller femoral head size, patients with developmental dysplasia, and certain implant designs. These results highlight the importance of patient and prosthesis selection in optimizing the outcome of hip resurfacing.

## Introduction

Conventional total hip replacement gives good outcome in older patients, but younger patients have higher revision rates (Boerre and Bannister 1993, Joshi et al. 1993, Dorr et al. 1994, Callaghan et al. 1997, Swedish Hip Arthroplasty Register Annual Report 2006). Resurfacing of the hip using a metal-on-metal, large-diameter bearing has theoretical advantages, particularly in younger patients. These include bone conservation, restoration of proximal femoral anatomy, low wear rates, and ease of future revision. Recent publications have reported promising results (Amstutz et al. 2004, Daniel et al. 2004, Back et al. 2005, Treacy et al. 2005, Girard et al. 2006, Pollard et al. 2006, Mont et al. 2007), especially in younger patients. There remain concerns, however, regarding increased risk of femoral neck fractures, metal ion release, and formation of pseudotumors (Shimmin and Back 2005, Grammatopoulos et al. 2009).

In Australia, hip resurfacing has been performed and recorded in the Australian Orthopaedic Association National Joint Replacement Registry (AOANJRR) since 1999. This report examines the 12,093 hip resurfacings reported to the Registry and evaluates risk factors for revision.

## Patients and methods

The AOANJRR started collection of data in September 1999. It was implemented in a stepwise manner, becoming fully operational on a national basis in 2002. All hospitals undertaking joint replacement surgery contribute data to the Registry. Cross-validation of procedures reported to the Registry with independently colleced health department data ensures that almost all hip procedures are recorded by the Registry. The present analysis includes all primary conventional and resurfacing total hip replacements recorded by the Registry up to and including December 2008 (147,422 hips in 129,992 patients and 12,093 hips in 10,489 patients, respectively).

### Statistics

The cumulative per cent revision (CPR) of primary total hip replacements was estimated at each of the first 8 years using the Kaplan-Meier method. Unadjusted CPR (with 95% confidence intervals) is reported. Hazard ratios (HRs) from Cox proportional hazards models, adjusting for age and sex where appropriate, were used to compare revision rates.

For each model, the assumption of proportional hazards was checked analytically. If the interaction between the predictor and the log of time was statistically significant in the standard Cox model, then a time varying model was estimated. Time points were selected based on the greatest change in hazard, weighted by a function of events. Time points were iteratively chosen until the assumption of proportionality was met; then the hazard ratios were calculated for each selected time period. In our results, if no time period is specified then the hazard ratio covers the entire follow-up period. Adjustment for bilaterality was not performed, as no bias in including bilateral replacements could be expected (Robertsson and Ranstam 2003).

All tests were two-tailed at the 5% level of significance. Analyses comparing outcomes of age, sex, and head size for resurfacing hip procedures, and conventional total versus resurfacing hip procedures, were performed on patients with primary diagnosis of osteoarthritis (OA) excluding revisions for infection.

## Results

### Conventional versus resurfacing

**Figure 1.**
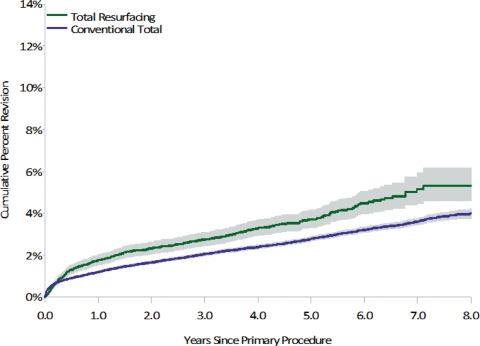
Cumulative percent revision of primary conventional total and total resurfacing hip replacement (primary diagnosis: OA, excluding infection).

The use of hip resurfacing increased from 6% of all primary total hip replacements in 2001 to 9% in 2005, but then gradually declined each subsequent year to 6% in 2008. Overall, hip resurfacing had a higher revision rate than conventional total hip replacement (age- and sex-adjusted HR = 1.4 (1.2–1.6)). The 8-year CPR for hip resurfacing was 5.3 (4.6–6.2), as compared to 4.0 (3.8–4.2) for conventional total hip replacement (Figure 1).

### Primary diagnosis

**Figure 2.**
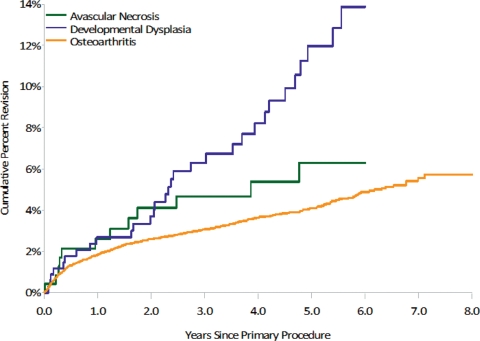
Cumulative percent revision of primary total resurfacing hip replacement, by primary diagnosis.

Nearly all patients undergoing primary resurfacing hip replacement had a primary diagnosis of osteoarthritis (OA) (94%). Patients with developmental dysplasia of the hip (DDH) had a higher rate of revision than those patients with OA (age- and sex-adjusted HR = 2.1 (1.4–3.1)) (Figure 2). The 5-year CPR for DDH patients was 12 (8–17) as compared to 4.1 (3.7–4.6) for OA patients. There was no difference in the rate of revision between avascular necrosis (AVN) and OA (age- and sex-adjusted HR = 1.6 (0.9–2.9)). The 5-year CPR for AVN patients was 6.3 (3.5–11). The 5-year CPR for inflammatory arthritis was 8 (4–18); however, the numbers are too small for a valid statistical comparison.

Age 55–64 vs. < 55 Entire period: HR = 1.3 (1.0–1.6), p = 0.04 Age 65–74 vs. < 55 Entire period: HR = 1.7 (1.2–2.5), p = 0.001 Age ≥ 75 vs. < 55 0–3 months: HR = 14.0 (5.7–35), p < 0.001 > 3 months: HR = 0.9 (0.1–6.1), p = 0.9

### Age, sex, and head size

**Figure 3.**
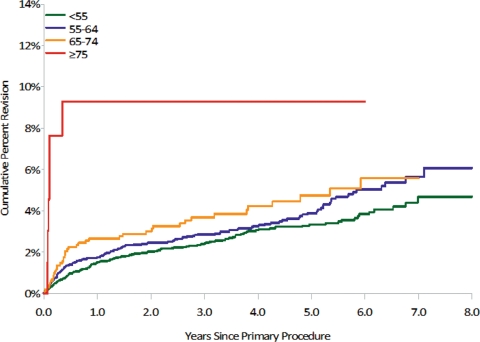
Cumulative per cent revision of primary total resurfacing hip replacement, by age (primary diagnosis: OA, excluding infection).

In 2008, 55% of the hip resurfacings were performed in patients aged less than 55 years, 38% were performed in patients aged 55–64, 7% in patients aged 65–74, and 1% in patients who were 75 years or older. This distribution has shown a slight increase in the proportion of younger patients (less than 65) in recent years. In patients with a diagnosis of OA, resurfacing procedures had an increasing risk of revision with increasing age (Figure 3). This is unlike conventional total hip replacement, where the risk of revision decreases with increasing age.

**Figure 4.**
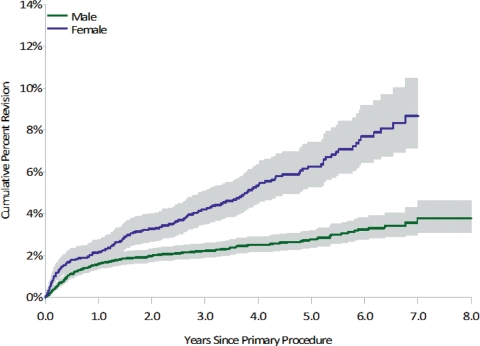
Cumulative per cent revision of primary total resurfacing hip replacement, by sex (primary diagnosis: OA, excluding infection).

**Figure 5.**
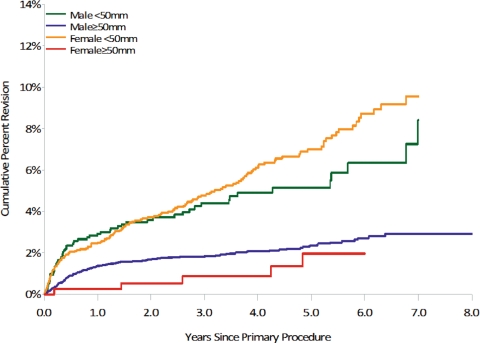
Cumulative per cent revision of primary total resurfacing hip replacement, by sex and femoral component head size (primary diagnosis: OA, excluding infection).

The proportion of hip resurfacings performed in females decreased steadily from 31% in 2002 to 20% in 2008. Females had a higher revision rate than males (age-adjusted HR = 2.2 (1.8–2.7)) (Figure 4). However, a higher proportion of females (65%) received head sizes less than 50 mm, and after adjusting for head size there was no difference in the rate of revision between males and females (age- and head-size adjusted HR = 1.0 (0.7–1.3)). For each sex, head sizes of less than 50 mm had a statistically significantly higher rate of revision than head sizes of 50 mm or greater (Figure 5).

< 50 mm vs. ≥ 50 mm: male, Entire period: HR = 2.4 (1.8–3.3), p < 0.001 female, Entire period: HR = 3.7 (1.7–7.9), p < 0.001 Female vs. male: < 50 mm, Entire period: HR = 1.2 (0.9–1.7), p = 0.2 ≥ 50 mm, Entire period: HR = 0.8 (0.4–1.7), p = 0.6

The 7-year outcome of primary resurfacing hip replacement in younger patients with a femoral head size of greater than 50 mm had a similar outcome to conventional total hip replacement at the same age. The 7-year CPR for hip resurfacing for those aged less than 55 years was 3.0 (2.2–4.2) and for those aged 55–64 it was 3.1 (2.3–4.2).

### Implant design

Since the introduction of modern hip resurfacing procedures, there has been a steady increase in the number of types of prostheses being used. In 2008, 13 types of resurfacing prostheses were used (Table 1). The Birmingham Hip Resurfacing (BHR) has remained the most frequently used prosthesis, making up 51% of all resurfacings in 2008. Its proportional use has, however, decreased from 96% in 2001. Up to the end of 2008, there were 9 resurfacing prostheses with over 100 procedures each recorded by the Registry. Of these, 3 prostheses had a statistically significantly higher revision rate than all other resurfacing procedures, as did 1 other prosthesis with 95 procedures. These prostheses were ASR (age- and sex-adjusted HR = 2.2 (1.7–2.9)), Durom (age- and sex-adjusted HR = 1.7 (1.2–2.4)), Cormet 2000 HAP (age- and sex-adjusted HR = 3 (1–5)) and Recap (age- and sex-adjusted HR = 3(1–5)). The ASR, Durom, and Recap prostheses were introduced to the Australian market after the BHR. Their outcome differs from that of other new prostheses with over 100 procedures, including the Adept and Mitch TRH, which had a CPR similar to that of the BHR at 3 years (Table 1).

**Table 1.** Annual cumulative per cent revision (with 95% CI) of primary total resurfacing hip replacement

Head component	Acetabular component	n (total)	1 year	3 years	5 years	7 years	8 years
ASR	ASR	1,073	3.6 (2.6–4.9)	6.0 (4.6–7.8)	8.7 (6.6–12)		
Adept	Adept	292	0.7 (0.2–2.7)	1.9 (0.7–5.1)			
BHR	BHR	8,427	1.5 (1.3–1.8)	2.5 (2.2–2.9)	3.6 (3.2–4.1)	4.8 (4.2–5.6)	5.0 (4.3–5.8)
Bionik	Bionik	119	4.3 (1.6–11)	6.7 (2.6–16.4)			
Conserve Plus	Conserve Plus	62	3.2 (0.8–12)	5.1 (1.7–15)	9.7 (4.1–22)	9.7 (4.1–22)	
Cormet	Cormet	192	1.6 (0.5–4.8)	3.8 (1.8–7.9)	5.3 (2.8–10)	6.0 (7.1–34)	
Cormet 2000 HAP	Cormet	95	6.3 (2.9–14)	8.4 (4.3–16)	9.5 (5.0–17)		
Cormet HAP BiCoat	Cormet	287	2.8 (1.3–5.8)	5.0 (2.6–9.5)			
Durom	Durom	767	3.0 (2.0–4.5)	4.7 (3.4–6.7)	6.7 (4.7–9.7)		
Icon	Icon	96	1.1 (0.2–7.9)	2.5 (0.6–9.6)			
Mitch TRH	Mitch TRH	534	1.4 (0.6–3.1)				
Recap	Recap	137	5.0 (2.3–11)	7.6 (3.8–15)			
Note: 2 resurfacing hip procedures using only a Conserve resurfacing head and no acetabular component have been excluded.							

The rate of revision for fracture in OA patients receiving hip resurfacing differed between the resurfacing prostheses. The BHR had the lowest risk of revision for fracture, with a CPR for fracture at 5 years of 1.2 (1.0–1.5) and a slight increase to 1.5 (1.2–1.9) at 8 years. When considering the prostheses with more than 100 procedures, 3 had a higher risk of revision for fracture than the BHR: the ASR (age- and sex-adjusted HR = 3 (2–5) p), the Durom (age- and sex-adjusted HR = 2 (1–4)), and the Recap (age- and sex-adjusted HR = 3 (1–9)).

### Revisions

Of the 12,093 hip resurfacings, 437 (3.6%) had been revised. Over half of the revisions were femoral-only revisions and one third were both femoral and acetabular revisions (Table 2). The most common reasons for revision were fracture, loosening/lysis, infection, and metal sensitivity (Table 3). The reason for revision changed with age, with an increase in revision for fracture with increasing age (1% of patients aged less than 55 years, 2% of patients aged between 55 and 64, 3% of patients aged between 65 and 74, and 9% of patients aged 75 and older). Females were not only revised more frequently for fracture than males (1.8% and 1.3%, respectively) but also for loosening/lysis (2.0% and 0.7%, respectively).

**Table 2.** Type of revision of primary total resurfacing hip replacement

Type of revision	n	%
Femoral only	252	58
THR (femoral/acetabular)	135	31
Acetabular only	37	9
Cement spacer	9	2
Removal of prosthesis	4	1
Total	437	100

**Table 3.** Reason for revision of primary total resurfacing hip replacement

Revision diagnosis	n	%
Fracture	172	39
Loosening/lysis	128	29
Infection	39	9
Metal sensitivity	28	6
Pain	23	5
Dislocation of prosthesis	14	3
Other	33	8
Total	437	100

## Discussion

With improvements in metal-on-metal bearing technology, hip resurfacing has gained popularity as an alternative to conventional total hip replacement in younger, active patients. Recent studies have shown promising results (Amstutz et al. 2004, Daniel et al. 2004, Back et al. 2005, Treacy et al. 2005, Girard et al. 2006, Pollard et al. 2006, Mont et al. 2007). It is generally accepted that younger patients with total hip replacements are at highest risk of revision (Boerre and Bannister 1993, Joshi et al. 1993, Dorr et al. 1994, Callaghan et al. 1997, Swedish Hip Arthroplasty Register Annual Report 2006). The Australian Joint Registry has reported an increase in the risk of revision (at 8 years) for patients less than 65 years with conventional total hip replacement (AOANJRR Annual Report 2009).

Our analysis shows that overall, primary hip resurfacing has a higher risk of revision than conventional total hip replacement after adjusting for age and sex. At 8 years, the CPR of hip resurfacing is 5.3 (4.6–6.2) as compared to 4.0 (3.8–4.2) for conventional total hip replacement. This difference was not apparent in patients aged less than 65 years with a femoral component greater than 50 mm. However, the difficulty in comparing this to all primary conventional total hip replacements is that most resurfacing procedures used a single prosthesis (BHR) that has been identified as having one of the lowest risks of revision. There are individual conventional total hip prostheses that the Registry reports as having a lower risk of revision than the reported overall risk of all conventional total hip prostheses (AOANJRR Annual Report 2009).

The rate of revision of resurfacing appears to be higher in females, and both sexes show an increasing revision rate with age. The higher revision rate in female patients can be accounted for by differences in the proportion of males and females who receive smaller sizes of femoral components. Head sizes of less than 50 mm have a higher risk of revision, but occur more frequently in the female population. These observations on femoral component size have been suggested previously. In a study of Conserve Plus hip resurfacings in 355 patients (400 hips), Amstutz et al. (2004) reported that female and male patients with smaller femoral component head sizes had more femoral loosening and radiolucencies. The authors concluded that femoral fixation is critical to long-term durability, and individuals with smaller head sizes have a smaller area available for cement fixation. The THARIES hip resurfacing also showed a lower survival rate for smaller heads (39%) than for larger component sizes (59%) at 11 years (Mai et al. 1996). However, other authors (Shimmin and Back 2005, Kim et al. 2008) have not reported any association between femoral head size and increased revision rate.

Even with head sizes of 50 mm and above, there was an increasing risk of revision with increasing age. This is presumably due to deteriorating bone quality. Thus, one might expect that women would have higher revision rates but our data suggest that being female is not a risk factor for revision of hip resurfacing. In fact, the relatively small number of females with head sizes of 50 mm and above 404 appear to have better survival rates than males (Figure 5). This observation may be due to differences in the selection process between the sexes, with many surgeons performing DEXA scans to assess bone density in females before performing hip resurfacing.

Patients with developmental hip dysplasia (DDH) had a higher risk of revision than those with primary osteoarthritis (OA). The 5-year CPR for DDH in patients aged less than 55 was more than 3 times that for OA (13 and 4, respectively). Schmalzried et al. (2005) reported that the outcome of hip resurfacing is dependent on the preoperative radiographic characteristics of the proximal femur (in terms of bone density, shape, limb length discrepancy, and neck shaft angle), with more normal morphology giving better outcome. The Registry data support these observations, with patients with abnormal anatomy—as in DDH—having a higher risk of revision than OA patients.

The 5-year CPR for AVN was higher than for OA (6 and 4, respectively) but this was not statistically significant. It has been reported that cysts in the femoral head are associated with early revision of hip resurfacing (Amstutz et al. 2004, Beaule et al. 2004). However, total hip replacement in young patients with AVN has generally been reported to have higher revision rates than in patients with primary OA. Early to medium-term revision rates of 39–57% have been reported (Chandler et al. 1981, Cornell et al. 1985, Saito et al. 1989, Mai et al. 1996, Callaghan et al. 1997, Beaule et al. 2004). The 5-year survival of hip resurfacing for AVN from our analysis (94%) is similar to that in other recently published series (Mont et al. 2006, Revell et al. 2006).

We found a difference in outcome related to the type of prosthesis used. The Durom, ASR, Cormet 2000 HAP, and Recap all had a higher rate of revision than other resurfacing prostheses. There may be many reasons for this, including differences in the patient population or surgeons, or in prosthesis designs. In our analysis, we considered all of these factors but found no patient-specific or surgeon-specific factors that might have contributed to the difference. Furthermore, this did not appear to be related to the later introduction of these prostheses (compared to the BHR) onto the Australian market, as there have been a number of other recent prostheses with a similar outcome to that of the BHR and other resurfacing prostheses. The difference in fracture rate between different prostheses may be related to the different designs and methods of preparing the femoral head.

### Interpretation

Although the Australian Joint Registry shows that, overall, hip resurfacings are revised more often than total hip replacements at up to 8 years, it also shows that hip resurfacing has a similar outcome to that of primary conventional total hip replacement in selected patients. Smaller femoral component sizes, increasing patient age, a diagnosis of developmental dysplasia of the hip, and some implant designs are risk factors for revision. The lowest cumulative per cent revision (3 at 7 years) was in patients aged less than 55 years with primary osteoarthritis and with femoral component sizes of ≥ 50 mm. It is too early to tell whether the perceived long-term benefits of hip resurfacing in this patient group will become apparent.
